# Control of spontaneous ovarian tumors by CD8+ T cells through NKG2D-targeted delivery of antigenic peptide

**DOI:** 10.1186/2045-3701-3-48

**Published:** 2013-12-20

**Authors:** Tae Heung Kang, Jayne Knoff, Benjamin Yang, Ya-Chea Tsai, Liangmei He, Chien-Fu Hung, T-C Wu

**Affiliations:** 1 Department of Immunology, College of Medicine, Konkuk University, Chungju, South Korea; 2 Department of Pathology, School of Medicine, Johns Hopkins University, Bldg. CRBII Rm. 309, 1550 Orleans Street, Baltimore, Maryland 21231, USA; 3 Department of Obstetrics and Gynecology, Johns Hopkins University, Baltimore, MD 21205, USA; 4 Department of Molecular Microbiology and Immunology, Johns Hopkins University, Baltimore, MD 21205, USA; 5 Department of Oncology, Johns Hopkins University, Baltimore, MD 21205, USA

**Keywords:** NKG2D, Ovarian cancer, Immunotherapy, T cell adoptive transfer, TgMISIIR-TAg transgenic mice

## Abstract

**Background:**

There is an urgent need to develop targeted therapies for the control of advanced stage ovarian cancer because it is the most deadly gynecologic cancer. Antigen-specific immunotherapy is a promising approach because of the potential of the immune system to specifically target tumors without the toxicity associated with traditional chemoradiation. However, one of the major limitations for antigen-specific cancer immunotherapy is the pre-existing immune tolerance against endogenous targeted tumor antigens that frequently evolves during carcinogenesis. Here, we described the creation of a therapeutic agent comprised of a tumor-homing module fused to a functional domain capable of selectively rendering tumor cells sensitive to foreign antigen-specific CD8+ T cell-mediated immune attack, thereby circumventing many aspects of immune tolerance. The tumor-homing module, NKG2D, specifically binds to NKG2D ligand that is commonly overexpressed in ovarian tumors. The functional domain is comprised of the Fc portion of IgG2a protein and foreign immunogenic CD8^+^ T cell epitope flanked by furin cleavage sites (R), which can be recognized and cleaved by furin that is highly expressed in the tumor microenvironment.

**Results:**

We show that this therapeutic chimeric protein specifically loaded antigenic epitope onto the surface of NKG2D ligand-expressing ovarian tumor cells, rendering ovarian tumors susceptible to antigen-specific CTL-mediated killing *in vitro.* Furthermore, we show that intraperitoneal administration of our therapeutic chimeric protein followed by adoptive transfer of antigen-specific CD8+ T cells generates potent antitumor effects and significant accumulation of antigen-specific CD8+ T cells in the tumor loci.

**Conclusions:**

Our findings have promise for bypassing immune tolerance to enhance cancer immunotherapy.

## Background

Advanced ovarian cancer is one of the most deadly malignancies and is responsible for the highest mortality rate among patients with cancers of the female reproductive system in the United States. Existing therapies for ovarian cancer, such as surgery and chemotherapy, have significant side effects and rarely result in long-term cures for patients with locally advanced or metastatic disease, although they can provide remission for several years [1,2]. The lack of curative treatments and high proportion of patients diagnosed with advanced disease underscores the urgent need to develop innovative and targeted therapies for the control of advanced stage ovarian cancer. Antigen-specific immunotherapy is a promising treatment strategy to enhance the control of advanced ovarian cancer, particularly to target minimal residual disease. Antigen-specific immunotherapy has the ability to harness the immune system to specifically target tumors without the toxicity associated with traditional chemoradiation (for review, see [3]). However, one of the major limitations for antigen-specific cancer immunotherapy is the inherited immune tolerance against endogenous tumor antigens targeted by the antigen-specific immunotherapy.

We have previously demonstrated that by selectively coating tumor cells with immunodominant foreign immunogenic CD8+ T cell peptide(s) recognized by pre-existing immunity, immune tolerance can be bypassed and pre-existing antigen-specific immunity was able to control the tumor [4]. We previously used a murine ovarian cancer model to design a therapeutic chimeric protein containing a tumor-homing module fused to a functional cargo domain. The tumor-homing portion of the therapeutic chimeric protein, an anti-mesothelin single chain variable fragment (meso-scFv), had high binding affinity to mesothelin [4], which is overexpressed by most ovarian cancers [5,6]. The cargo domain of the therapeutic chimeric protein was comprised of the Fc portion of the IgG2a protein and a MHC class I-restricted foreign immunogenic CTL epitope, OVA, flanked by furin cleavage sites. We showed that the antigenic CTL epitope could be loaded onto tumor cells following selective cleavage of the amino acid sequence RVKR by furin. Many tumors, including ovarian tumors (for review, see [7]), highly express furin on the cell surface and in the extracellular space [8-12]. We showed that meso-scFv preferentially delivered OVA to mesothelin-expressing ovarian cancer cells, where cleavage by furin released the foreign immunogenic CTL epitope to be loaded on MHC class I molecules of tumor cells [4]. Notably, this rendered tumor cells susceptible to OVA-specific CTL-mediated killing, both *in vitro* and *in vivo *[4]. Thus, specific molecules expressed by ovarian tumors, such as mesothelin, can be used as targets for the delivery of antigeneic CTL peptides. The encouraging results from these studies in the ovarian tumor model warrant further exploration of the molecular targets specifically expressed by tumors for our therapeutic approach.

The identification of surface molecules that are specifically expressed in tumor cells but not in normal cells becomes important to facilitate the specific delivery of antigenic CTL peptides to tumor loci. For example, multiple NKG2D ligands have been identified and are known to be upregulated in transformed, infected, and/or stressed cells but not in substantial amounts in healthy adult cells [13]. The upregulation of NKG2D ligands may be due to cellular and genotoxic stresses, such as excessive proliferation, heat shock, or oxidative stress. Consequently, NKG2D ligands are highly expressed in various tumors of different origins to varying degrees [14]. Thus, protein or antigenic peptides potentially can be linked to NKG2D for their specific delivery to tumor loci. We have previously successfully employed NKG2D to specifically deliver a linked molecule of interest to tumor loci [15].

In the current study, we created a chimeric protein consisting of NKG2D linked to the Fc domain of IgG2a and the OVA CTL peptide flanked by a furin cleavage site to form NKG2D-Fc-RO. We showed that ovarian tumor cells that express the NKG2D ligand Rae-1 can be bound by the chimeric NKG2D-Fc-RO protein and present the OVA CTL peptide through MHC class I molecules. In addition, binding of NKG2D-Fc-RO to tumor cells renders them susceptible to OVA-specific CD8+ T cell-mediated killing. More importantly, we showed that intraperitoneal injection of the chimeric NKG2D-Fc-RO protein is capable of generating potent therapeutic antitumor effects against NKG2D ligand expressing spontaneous ovarian tumor following adoptive transfer of OVA-specific CD8+ T cells. This study serves as a foundation for future therapies utilizing targeted coating of tumor cells with antigenic peptide.

## Results

### Generation and characterization of the NKG2D-Fc-RO chimeric protein

**Figure 1:**
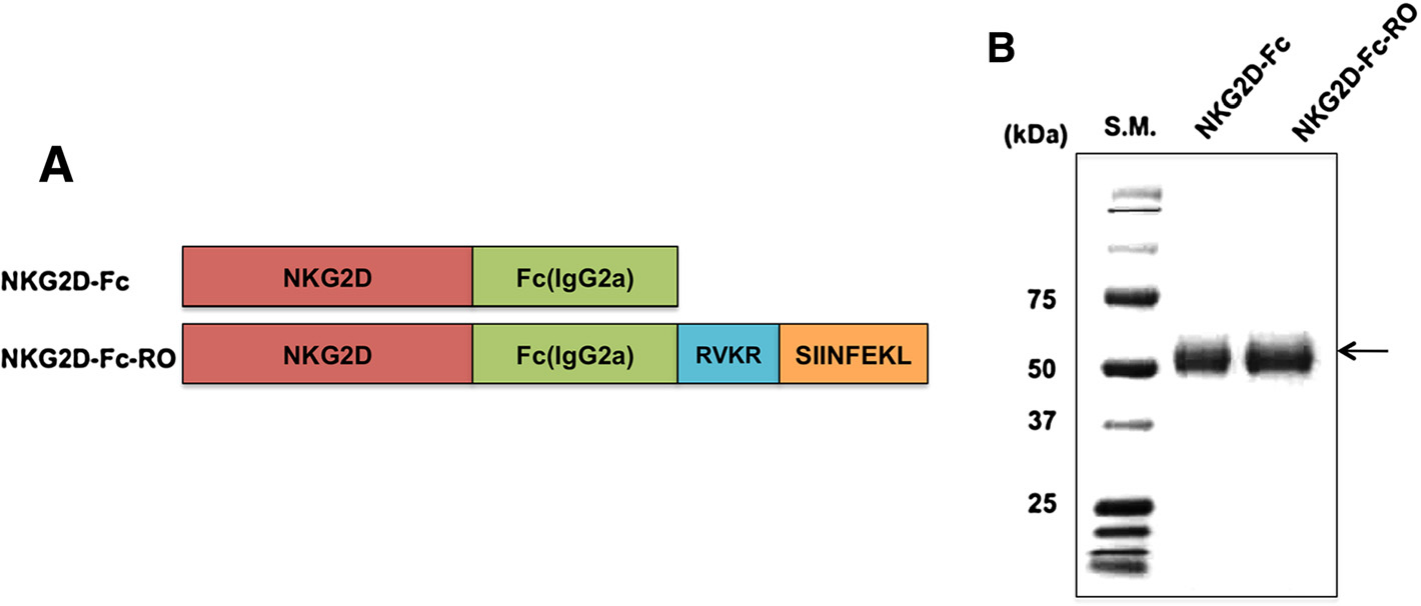
**Generation and characterization of therapeutic chimeric proteins. (A)** Schematic diagram of chimeric proteins containing NKG2D, Fc protein (IgG2a), furin cleavage site (RVKR), and ovalbumin peptide (SIINFEKL). **(B)** Gel electrophoresis of isolated proteins from furin-deficient FD11 cells transfected with the NKG2D-Fc and NKG2D-Fc-RO. Protein was isolated from supernatant of cultured cells and then characterized by sodium dodecyl sulfate-PAGE and Coomassie Brilliant Blue staining.

We created a chimeric protein consisting of NKG2D conjugated with Fc (IgG2a) protein and containing OVA peptide (O) flanked by a furin cleavage site (R) (NKG2D-Fc-RO). Figure 1A shows the schematic diagram of the chimeric NKG2D-Fc-RO construct as well as the control NKG2D-Fc protein without OVA peptide and furin cleavage sites. We have successfully isolated the NKG2D-Fc-RO and NKG2D-Fc proteins from the supernatant of furin-deficient CHO-based cell line FD11 stably transduced with the expression vector and used SDS-PAGE to assess their size and purity, as shown in Figure 1B. The stained gel shows bands of high purity and of the expected size for both proteins. Because the difference in molecular weight between NKG2D-Fc-RO and NKG2D-Fc is only 12 amino acids, the proteins show similar migration on the SDS-PAGE gel.

### The NKG2D-Fc protein binds to murine ovarian tumor cells expressing murine NKG2D ligands

**Figure 2:**
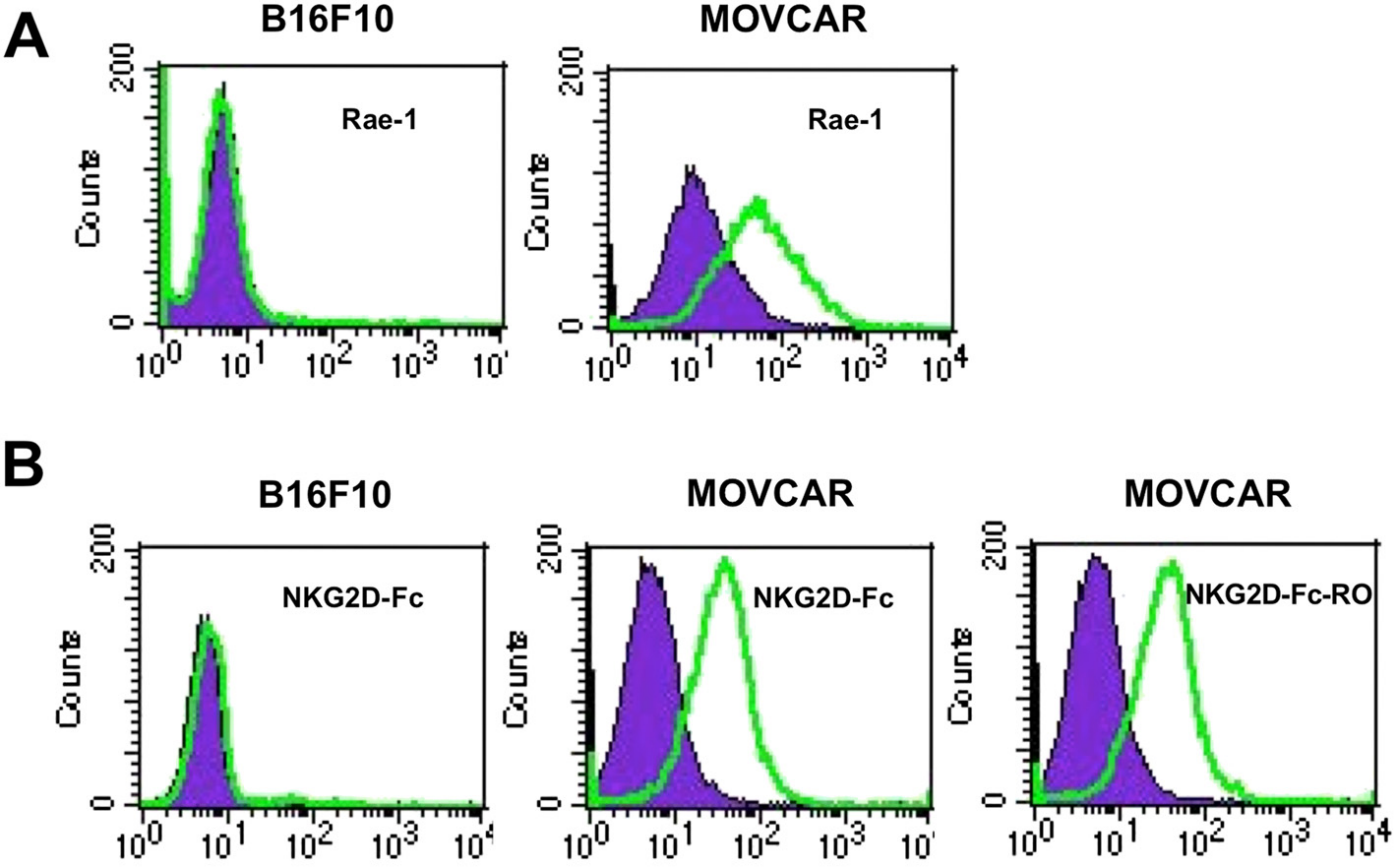
**Characterization of the binding of NKG2D-Fc and NKG2D-Fc-RO to NKG2D ligand expressed by MOVCAR tumor cells using flow cytometry. (A)** Representative flow cytometry analysis of antibody bound to Rae-1 (clear histogram) or isotype (shaded histogram) with B16F10 (negative control) and MOVCAR cells. **(B)** Purified proteins Fc (shaded histogram), NKG2D-Fc (clear histogram) and NKG2D-Fc-RO (clear histogram) were incubated with either B16F10 or MOVCAR cells and then stained with phycoerythrin (PE)-labeled antimouse Fc antibody.

To determine if the purified NKG2D-Fc-RO protein could bind to murine NKG2D ligand-expressing tumor cells, we selected a Rae-1 (a murine NKG2D ligand)-expressing ovarian tumor cell line (MOVCAR) and one Rae-1-negative tumor cell line (B16F10) for our studies (Figure 2A). As shown in Figure 2B, both NKG2D-Fc-RO and NKG2D-Fc bound to the NKG2D ligand-expressing murine ovarian tumor cells, MOVCAR, but not to the Rae-1-negative B16F10 tumor cells. In comparison, the MOVCAR cells incubated with purified Fc protein alone did not demonstrate specific binding (Figure 2B). This is consistent with previous reports that cells engineered to express the extracellular domain of murine NKG2D are capable of recognizing and binding to cells NKG2D ligand-expressing cells, including Rae-1-expressing cells [16]. Taken together, these data indicate that NKG2D-Fc and NKG2D-Fc-RO are able to bind to NKG2D ligand-expressing tumor cells.

### The binding of NKG2D-Fc-RO to NKG2D ligand-expressing tumor cells leads to MHC class I presentation of OVA peptide to OVA-specific CD8^+^ T cells

**Figure 3:**
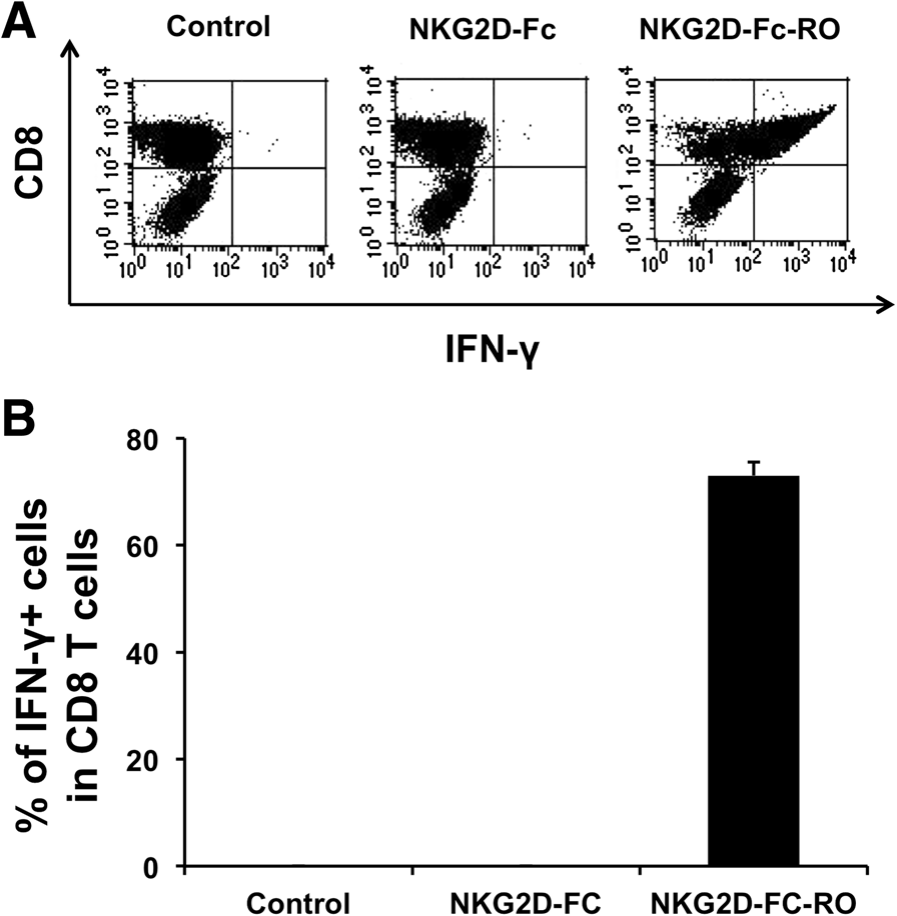
**Major histocompatibility complex class I presentation of ovalbumin (OVA) peptide to OVA-specific CD8** **+** **T cells by MOVCAR cells treated with NKG2D-Fc-RO. (A)** Flow cytometry characterization of OVA-specific CD8+ T-cell activation by MOVCAR cells treated with different chimeric proteins followed by incubation with 2 × 10^5^ OVA-specific cytotoxic CD8+ T lymphocytes (CTLs). OVA-specific CD8+ T-cell activation was determined by CD8 and IFN-γ staining. **(B)** Representative bar graph depicting the % of IFN-γ-secreting OVA-specific CD8+ T cells out of total OVA-specific T cells (mean ± SD).

We further determined if the binding of NKG2D-Fc-RO to MOVCAR cells would enable MHC class I presentation of OVA peptide to OVA-specific CD8+ T cells and result in the activation of OVA-specific CD8^+^ T cells. As shown in Figure 3, MOVCAR cells incubated with NKG2D-Fc-RO led to the greatest activation of OVA-specific CD8^+^ T cells compared to MOVCAR cells incubated with NKG2D-Fc. Thus, these results suggest that NKG2D-Fc-RO specifically binds MOVCAR and facilitates MHC class I presentation of OVA peptide to activate OVA-specific CD8+ T cells.

### The binding of NKG2D-Fc-RO to NKG2D ligand-expressing tumor cells renders tumor cells susceptible to OVA-specific CD8^+^ T cell-mediated killing

**Figure 4:**
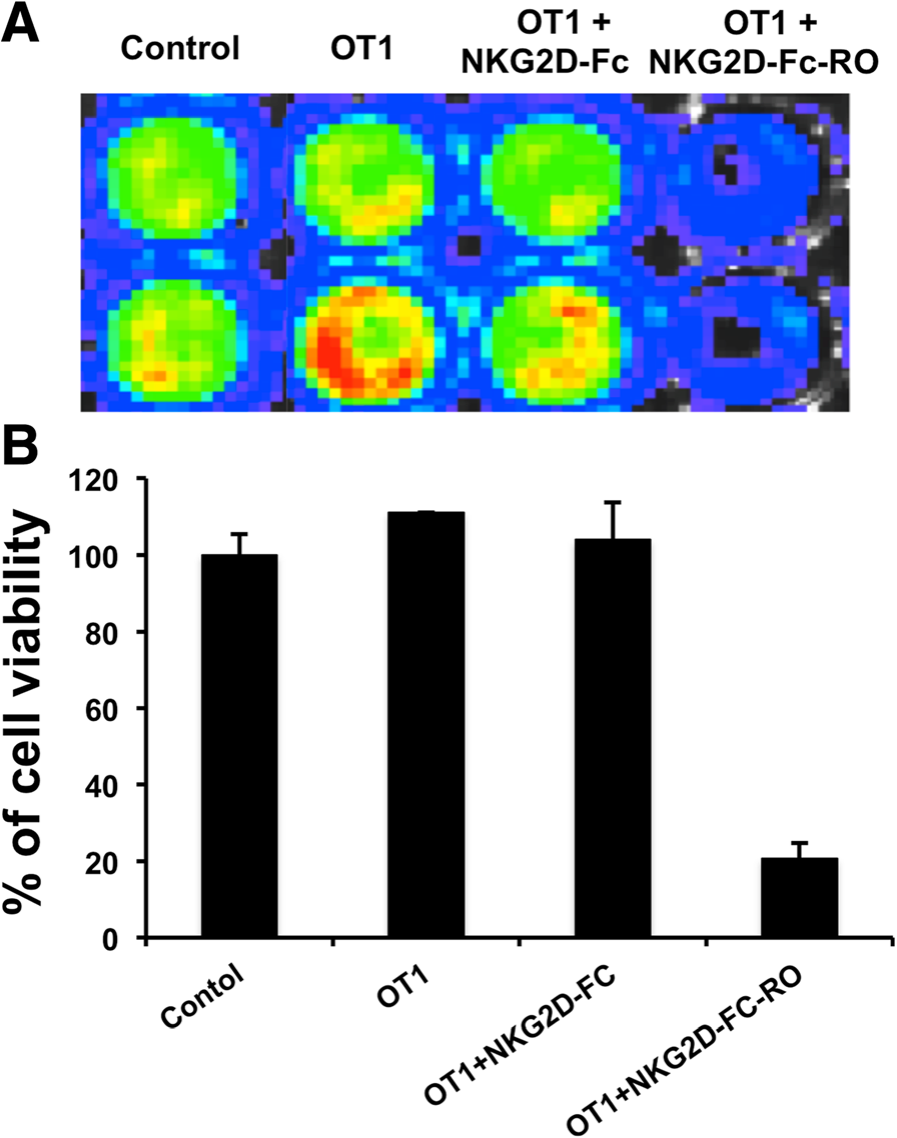
**Characterization of the CTL-mediated killing of MOVCAR tumor cells treated with the various chimeric proteins. (A)** Representative luminescence imaging of *in vitro* OVA-specific CTL killing of luciferase-expressing MOVCAR cells treated with 0.5 μg/ml NKG2D-Fc or NKG2D-Fc-RO. Luciferase-expressing MOVCAR cells treated with chimeric proteins were later incubated with 2 × 10^4^ OVA-specific CD8+ T cells (OT-1 T cells). CTL-mediated tumor cell death was determined by decreasing luminescence activity. **(B)** Bar graph depiction of tumor cell viability after treatment with chimeric protein and/or OT-1 T cells (mean ± SD) (data representative of two experiments).

We then determined if binding of NKG2D-Fc-RO to MOVCAR tumor cells would render luciferase-expressing MOVCAR tumor cells susceptible to OVA-specific CD8^+^ T cell killing. As shown in Figure 4, in the presence of OVA-specific CD8+ T cells, OT-1 cells, luciferase-expressing MOVCAR incubated with NKG2D-Fc-RO had the greatest amount of OVA-specific CTL-mediated tumor cell death, as measured by a significant reduction in luminescence activity among different experimental groups. Luciferase-expressing MOVCAR tumor cells incubated with NKG2D-Fc did not render tumor cells more susceptible to OVA-specific CD8+ T cell mediated killing. These data suggest that the binding of NKG2D-Fc-RO to tumor cells renders bound tumor cells susceptible to OVA-specific CTL killing.

### Treatment of spontaneous tumor-bearing MISIIR mice with chimeric protein NKG2D-FcRO leads to potent antitumor effects

**Figure 5:**
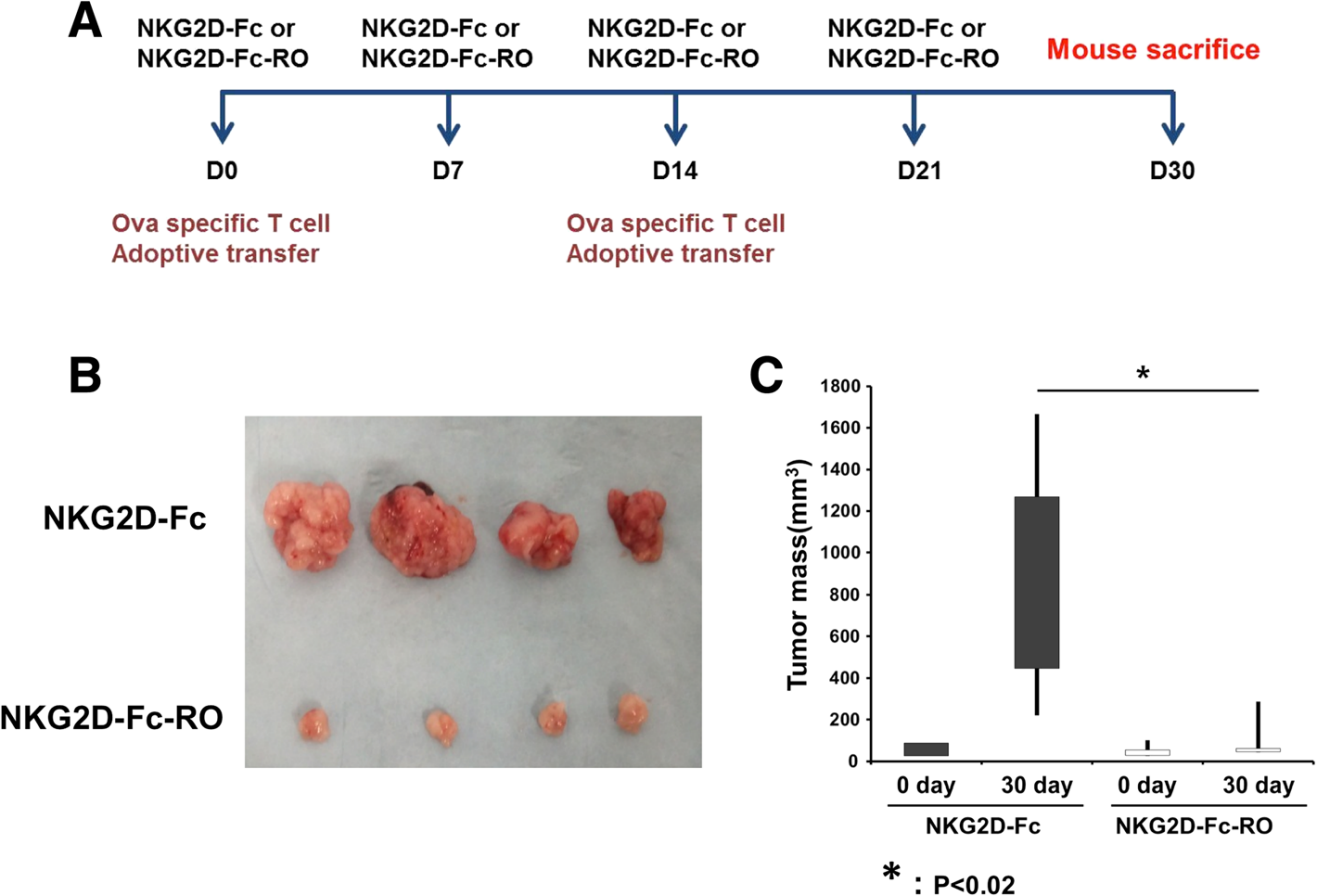
**Characterization of therapeutic antitumor effects and antigen-specific CD8+ T cell in ovarian tumors following intraperitoneal injection of chimeric NKG2D-Fc-RO protein.** 10-week-old tumor-bearing MISIIR mice were treated with 20 μg/mouse of NKG2D-Fc or NKG2D-Fc-RO protein every week for four weeks and 2.5x10^6^ OT-1 CD8+ T cells were injected intraperitoneally every other week two times. Mice were sacrificed 1 week after the last treatment. Ovarian tumors were harvested for measurement. **A**. Schematic diagram of the therapeutic regimens. **B** Representative ovarian tumors treated with NKG2D-Fc or NKG2D-Fc-RO protein. **C**. Bar graph of the tumor masses of mice treated with NKG2D-Fc or NKG2D-Fc-RO protein.

We next examined the efficacy of treatment with the chimeric protein including the OVA antigen, NKG2D-Fc-RO, in tumor-bearing TgMISIIR-TAg transgenic mice. We have previously shown that MISIIR transgenic mice spontaneously develop ovarian tumor approximately 10 weeks after birth [17]. Mice with similar sizes of ovarian tumors were selected to receive treatment with NKG2D-Fc or NKG2D-Fc-RO and OVA-specific OT-1 CD8+ T cells injected intraperitoneally, as outlined in the treatment schedule depicted in Figure 5A. We found that female TgMISIIR-TAg transgenic mice treated with NKG2D-Fc-RO had significantly reduced tumor mass after 30 days compared to those treated with NKG2D-Fc (Figure 5B and C). These data suggest that intraperitoneal injection of the chimeric NKG2D-Fc-RO protein is capable of generating potent therapeutic antitumor effects against NKG2D ligand expressing ovarian tumors following adoptive transfer of OVA-specific CD8+ T cells.

### *S*pontaneous ovarian tumor-bearing TgMISIIR-TAg transgenic mice treated with chimeric NKG2D-Fc-RO protein in conjunction with adoptive transfer of OVA-specific CD8+ T cells leads to accumulation of OVA-specific CD8+ T cell in the tumor loci

**Figure 6:**
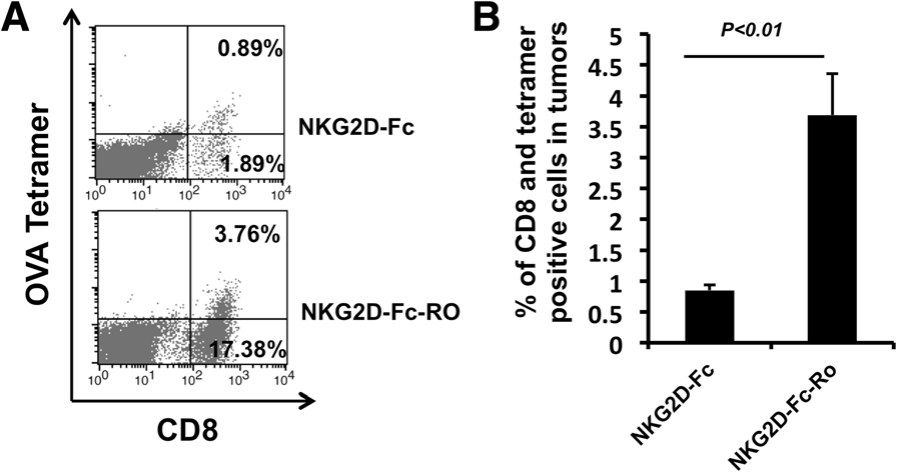
**Characterization of OVA-antigen specific CD8+ T cell in the tumor infiltrating lymphocytes derived from the spontaneous ovarian tumors following intra-peritoneal injection of chimeric NKG2D-Fc-RO protein.** 10-week-old tumor-bearing MISIIR mice were treated with 20 μg/mouse of NKG2D-Fc or NKG2D-Fc-RO protein every week for four weeks and 2.5x10^6^ OT-1 CD8+ T cells were injected intraperitoneally every other week two times. Mice were sacrificed 1 week after the last treatment. Ovarian tumors were harvested and tumor infiltrating lymphocytes were characterized for OVA-specific CD8+ T cells using OVA peptide-loaded H-2K^b^ tetramer staining. **A**. Representative flow cytometry analysis for the characterization of OVA-specific CD8+ T cells in the tumors. **B**. Bar graph depicting the percentage of OVA-specific CD8+ T cells in total TILs (mean ± SD).

In order to determine if treatment of the spontaneous ovarian tumor-bearing mice with the chimeric NKG2D-Fc-RO protein in conjunction with adoptive transfer of OVA-specific CD8+ T cell can lead to the expansion of OVA-specific CD8+ T cells in the ovarian tumor loci, we characterized the presence of OVA-specific CD8+ T cell among the TILs derived from the ovarian tumors using OVA CTL peptide loaded H-2Kb tetramer labeling. As shown in Figure 6A and B, we observed that tumor-bearing TgMISIIR-TAg transgenic mice treated with NKG2D-Fc-RO had a significantly higher percentage of OVA-specific CD8+ T cells among TILs compared to those treated with NKG2D-Fc. These data indicate that treatment with NKG2D-Fc-RO may target the linked antigenic peptide to NKG2D ligand expressing tumor cells and promote the presentation of antigenic peptide by tumor cells, which enhances the expansion of OVA-specific CD8+ T cells in the tumor loci.

## Discussion

In this study, we demonstrated that a chimeric protein, NKG2D-Fc-RO, was able to bind to murine NKG2D ligand-expressing ovarian tumor cells in vitro. Furthermore, we were able to use the NKG2D-Fc protein to deliver a model ovalbumin antigenic peptide to NKG2D ligand-expressing ovarian tumor cells to facilitate MHC class I presentation of the antigenic peptide. Additionally, we demonstrated that OVA CTL peptide delivered by NKG2D-Fc-RO to tumor cells rendered tumor cells susceptible to OVA-specific CTL-mediated killing. More importantly, we demonstrated that intraperitoneal injection of the chimeric NKG2D-Fc-RO protein in spontaneous ovarian tumor bearing mice significantly expanded OVA-specific CD8+ T cells at the tumor loci and resulted in the control of spontaneous ovarian tumors. Thus, our study demonstrated that chimeric proteins containing NKG2D are capable of specifically targeting NKG2D ligands, often found to be overexpressed on tumor cells, making the NKG2D-ligand system an exploitable receptor-ligand system to bring potentially immune-modulating or therapeutic molecules to the tumor site.

The data generated from our current study is consistent with data generated from previous studies using NKG2D as a carrier to deliver protein/antigen of interest to NKG2D ligand expressing tumor cells in tumor bearing mice for the development of therapeutic approaches. Previously, we used NKG2D to specifically deliver IL-2 to Rae-1 expressing TC-1 tumors in tumor-bearing mice. We found that by linking NKG2D to IL2, we were able to specifically deliver IL-2 to the tumor location, enhancing antigen-specific T-cell immune response and controlling tumor growth [15].

Our approach successfully exploited an NKG2D ligand expressed on tumor cells, to specifically deliver a therapeutic agent. Our findings warrant the examination of other molecules that are also uniquely expressed or overexpressed on the surface of tumor cells, but not on normal cells. For example, mesothelin is a commonly overexpressed protein on many human cancer cells, including ovarian [5,6] and pancreatic cancer [5]. Indeed, we have previously utilized mesothelin for targeting ovarian tumor cells [4]. Other conceivable molecules for targeting ovarian cancer cells include follicle-stimulating hormone receptor (FSHR) [17], intercellular adhesion molecule 1 (ICAM-1) [18], Müllerian inhibiting substance type II receptor (MISIIR) [19], and human epidermal growth factor receptor 2 (HER2) [20]. Leading-edge technologies including deep sequencing, gene and protein microarrays, proteomics, have the capacity to identify more potential candidate targets in the near future that may be used to deliver specific molecules to the tumor loci using a receptor-ligand system or an antibody-antigen system.

One potential limitation for the clinical translation of the proposed approach is antibody generation, specifically against the chimeric protein. If antibodies are generated against the chimeric protein, subsequent challenges may be affected. Furthermore, there are concerns that the chimeric protein may be allergenic and could be rapidly eliminated due to immune responses against the chimeric protein. Therefore, as a step towards clinical translation, it will be important to use the human counterparts for the murine NKG2D, Fc, R, and O of this study to create a clinical grade reagent that elicits limited immune responses against the therapeutic chimeric protein. It will also be important to test the binding ability of the human NKG2D-Fc-RO to various human ovarian cancer cell lines before considering generating a clinical grade reagent for clinical trials.

## Conclusions

In summary we have successfully developed a strategy to specifically target a therapeutic chimeric protein to tumor loci, which elicits potent tumor-targeted killing through antigen-specific CD8+ immune responses. This strategy may provide a platform for the delivery various anti-cancer molecules to the tumor loci as well as for coating tumor cells to circumvent immune tolerance in order to generate therapeutic antitumor effects. Furthermore, this strategy can be used in conjunction with active immunization against foreign antigenic peptide that is incorporated in the chimeric protein in order to enhance the antigen-specific CD8+ T cell immune responses. Since NKG2D ligands are highly expressed in many different tumor cells, our strategy represents a potentially useful approach for delivering molecules of interest to tumor loci for the control of different kinds of tumors for future clinical translation.

## Methods

### Mice

Female TgMISIIR-TAg transgenic mice were obtained by breeding female C57BL/6 mice with male TgMISIIR-TAg transgenic mice [21]. The male MISIIR-TAg transgenic mice were obtained from Fox Chase Cancer Center (Philadelphia, PA, USA) [21]. Female TgMISIIR-TAg transgenic mice 10 weeks of age were used in the experiments. These mice spontaneously developed ovarian tumors with complete tissue penetration. All animals were maintained under specific pathogen-free conditions, and all procedures were performed according to approved protocols and in accordance with recommendations for the proper use and care of laboratory animals by Johns Hopkins University Animal Care and Use Committee (protocol MO11M398).

### Cells

The MOVCAR (mouse ovarian carcinoma) cell line was obtained from Fox Chase Cancer Center (Philadelphia, PA, USA). It was derived from ascites of a TgMISIIR-TAg transgenic mouse with ovarian tumors [22]. All cell lines were maintained in RPMI 1640 complete medium supplemented with 10% heat-inactivated FBS (HyClone), 1% non-essential amino acid, 2 mM l-glutamine, 50 U/ml penicillin, 50 mg/ml streptomycin, and 5 mM 2-ME (Invitrogen Life Technologies). To generate the MOVCAR-luc cell line, luciferase expressing MOVCAR tumor cells (MOVCAR-luc) were generated by transducing the MOVCAR cells with retrovirus containing luciferase, pLuci-thy1.1, and flow cytometry sorting following the previously described protocol [23]. OVA-specific CD8+ T cells (OT-1) were produced via the stimulation of splenocytes obtained from OT-1 transgenic mice with irradiated EG.7 cells in the presence of interleukin-2 (20 IU/ml, Pepro-Tech, Rock Hill, NJ).

### Plasmid DNA constructs and preparation

Pfuse-NKG2D-Fc was described previously [15]. To generate pFuse-NKG2D-Fc-RO the oligos CTAGACGGGTGAAGCGGAGTATAATCAACTTTGAAAAACTGTAAC and TCGAGTTACAGTTTTTCAAAGTTGATTATACTCCGCTTCACCCGT were annealed and cloned into XbaI/Xho sites of Pfuse-NKG2D-Fc.

### Transfection and protein purification

For purification of the NKG2D-FC and NKG2D-FC-RO, 50 μg of plasmid was transfected into 1 × 10^7^ FD11 cells in T-150 flask using Lipofectamin 2000 (Invitrogen Corp., Carlsbad, CA, USA). After 3 days, cell culture media was accumulated, filtered using 0.22 μm syringe filter (Millipore, Billerica, MA, USA) and concentrated with Amicon cut-off 50 kDa Ultra -15 (Millipore, Billerica, MA, USA). Concentrated recombinant protein containing media was applied to a HiTrap Protein G HP column (GE Healthcare) following the vendor’s protocol. Protein concentrations were determined by the Coomassie Plus protein assay (Pierce, Rockford, IL, USA) and purity was estimated by SDS polyacrylamide gel electrophoresis.

### Cell staining and flow cytometry analysis

For flow cytometry analysis, tumor cells were stained with Rae-1 antibody (BD Bioscience), or 0.5 μg of NKG2D-Fc and NKG2D-Fc-RO respectively and PE-conjugated anti-mouse antibody was used as a detection antibody (BD Bioscience). The percentage of OVA-specific IFN-γ secreting CD8^+^ T cells was determined using intracellular cytokine staining and FACScan analysis with CELLQuest software (Becton Dickinson Immunocytometry System, Mountain View, USA).

### OVA-specific T cell activation and In vitro cytotoxicity assay

For T cell activation, MOVCAR cells were added to 48-well plates at a dose of 1 × 10^5^ cells/well and incubated with 0.5 μg/ml of proteins. Eighteen hours later, treated tumor cells were incubated with 2 × 10^5^ OVA-specific cytotoxic T cells (CTL). One day after activation, IFN-γ-secreting OVA-specific CTLs were identified by intracellular cytokine staining and analyzed by flow cytometry analysis. For the *in vitro* cytotoxicity experiment, 1 × 10^4^ of luciferase-expressing MOVCAR (MOCAR-luc) cells were incubated with 0.5 μg/ml of one of the various proteins on 96-well plate for 6 hours and treated with 2 × 10^4^ OVA-specific CTLs. The degree of CTL-mediated killing of the tumor cells was measured by the IVIS luminescence imaging system series 2000 as described previously [4].

### In vivo tumor treatment experiments

Tumor growth was assessed in 10-week-old female TgMISIIR-TAg transgenic mice by visual inspection following open surgery. Mice (5 per group) with similar sized ovarian tumors were selected to receive treatment with 20 μg/mouse of NKG2D-Fc or NKG2D-Fc-RO protein every week for four weeks. Prior to treatment, mice to be treated with NKG2D-Fc had an average tumor size of 55.02 ± 21.18 mm^3^ and mice to be treated with NKG2D-Fc-RO had an average tumor size of 52.36 ± 27.04 mm^3^. Mice were intraperitoneally injected with 2.5x10^6^ OT-1 CD8+ T cells twice, every other week. Female TgMISIIR-TAg transgenic mice without treatment were also included for comparison. Mice were sacrificed 1 week after the last treatment. Ovarian tumors were harvested for measurement. Tumor infiltrating lymphocytes (TILs) in the ovarian tumors were characterized for the presence of OVA-specific CD8+ T cells using OVA peptide-loaded H-2K^b^ tetramer staining and CD8 staining.

### Statistical analysis

The data presented in this study are representative of at least two experiments performed, and are expressed as means ± standard deviation (S.D.). The number of samples in each group for any given experiment was >3. Results for intracellular cytokine staining with flow cytometry analysis and tumor treatment experiments were evaluated by analysis of variance (one-way ANOVA) and the Tukey-Kramer multiple comparison test. Comparisons between individual data points were performed using Student’s t-test. Statistical analysis was performed with GraphPad Prism 4.0 software and a p value < 0.05 was considered significant.

## Abbreviations

R: Furin cleavage site; CTL: Cytotoxic T lymphocyte; Meso: Mesothelin; ScFv: Single chain variable fragment; OVA: O – ovalbumin; MHC: Major histocompatibility complex; MOVCAR: Mouse ovarian carcinoma cell line; OT-1: OVA-specific CD8+ T cells; MISIIR: Müllerian inhibiting substance type II receptor; FSHR: Follicle-stimulating hormone receptor; ICAM-1: Intercellular adhesion molecule 1; HER2: Human epidermal growth factor receptor 2.

## Competing interests

The authors declare that they have no competing interests.

## Authors’ contributions

THK, CFH and TCW conceived and designed experiments and interpreted data. THK, YCT and LH performed experiments. JK, BY, CFH and TCW wrote the manuscript. All authors read and approved the final manuscript.
